# Unique classification of parathyroid dysfunction in patients with transfusion dependent thalassemia major using Nomogram-A cross sectional study

**DOI:** 10.1016/j.amsu.2019.07.016

**Published:** 2019-07-11

**Authors:** Hafsa Majid, Lena Jafri, Sibtain Ahmed, Jamsheer Talati, Bushra Moiz, Aysha Habib Khan

**Affiliations:** a Section of Clinical Chemistry, Department of Pathology and Laboratory Medicine, Aga Khan University, Stadium Road, P.O. Box 3500, Karachi, 74800, Pakistan; b Department of Surgery, Aga Khan University, Stadium Road, P.O. Box 3500, Karachi, 74800, Pakistan; c Section of Hematology, Department of Pathology and Laboratory Medicine, Aga Khan University, Stadium Road, P.O. Box 3500, Karachi, 74800, Pakistan

**Keywords:** β thalassemia major (β–TM), Parathyroid hormone (PTH), Intact parathyroid hormone (iPTH), Primary hypoparathyroidism, Sub-clinical hypoparathyroidism, Vitamin D deficiency (VDD), Secondary hyperparathyroidism (SHPT)

## Abstract

**Introduction:**

Hypoparathyroidism is a rare complication of iron overload in patients with transfusion dependent β thalassemia major (β-TM). We aim to determine the prevalence of parathyroid dysfunction in patients with β-TM.

**Methods:**

Diagnosed cases of transfusion dependent β-TM between 5 and 17 years of age were recruited from outpatient clinics of a non-profit organization in Karachi, Pakistan. Blood and urine samples were collected in fasting to determine Ca, P, Alb, Mg, Cr 25OHD and iPTH. Patients were grouped on the basis of upper and lower levels of Ca, 25OHD and iPTH for assessing parathyroid dysfunction into primary hypoparathyroidism [*low calcium (Ca) & intact parathyroid hormone (iPTH)*], sub-clinical hypoparathyroidism [*low iPTH and 25 hydroxy vitamin D (25OHD), low/normal Ca],* normal functioning parathyroid gland [*Normal Ca, iPTH and 25OHD*] and secondary hyperparathyroidism [*high iPTH, low/normal Ca and/or 25OHD*]. Using PTH nomogram subject specific expected PTH (maxPTH) was calculated. Difference between maxPTH and measured iPTH was determined to assess the utility of nomogram in identifying parathyroid gland dysfunction. The statistical analysis was performed using the Statistical Package of Social Sciences (SPSS) version 20.

**Results:**

Median age of patients was 11 years (13–7) with males being 54.2% (n = 205).

Based on Ca, 25OHD and iPTH, primary hypoparathyroidism was identified in 3.4% (n = 13) [median iPTH 11.3 pg/ml (12.6–7)], 52.3% (n = 192) had subclinical hypoparathyroidism [iPTH 40.4 pg/ml (52.7–28.7)], and 34% (n = 125) were identified as secondary hyperparathyroidism [iPTH 88.6 pg/ml (116–74.7)]. Normal response to Ca & 25OHD was seen in 10.6% (n = 39) [iPTH 44.2 pg/ml (53.8–33.4)] patients. High phosphorous was present in all groups. Difference between maxPTH & iPTH was highest in primary hypoparathyroidism, followed by subclinical and secondary hyperparathyroidism.

**Conclusion:**

Nomogram by Harvey et al. identify low secretion capacity of parathyroid gland that correlated with biochemical classification of patients. It requires clinical validation before using in clinical practice for assessing parathyroid dysfunction.

## Highlights

• Prevalence of parathyroid dysfunction in transfusion dependent β-TM using biochemical markers and difference between measured and maxPTH using nomogram by Harvey et al. yield better diagnostic yield.
• Primary hypoparathyroidism developed at an early age as compare to that reported in literature.
• Subclinical hypoparathyroidism can be determined by applying this approach.
• In developing countries screening for parathyroid dysfunction should start from 9 years onwards rather than 16 years.
• Nomogram by Harvey et al. identify low secretion capacity of parathyroid gland that correlated with biochemical classification of patients.

## 1 Introduction

Parathyroid dysfunction notably hypoparathyroidism, is a rare complication of iron overload in patients with transfusion dependent β thalassemia major (β-TM) [1,2]. Manifestations of hypoparathyroidism include tetany, seizures, carpopedal spasms, and paresthesia, which may or may not be accompanied by hypocalcemia [3]. It is recommended to screen for parathyroid dysfunction periodically, when other iron overload associated complications occur, as ferritin levels do not provide a reliable index of parathyroid function [4].

The availability of different treatment modalities has increased quality and life expectancy of patients with thalassemia worldwide [5]. However, in Pakistan due to inadequate management; treatment related complications continue to add to morbidity in these patients.

In a cross sectional survey of patients with transfusion dependent β-TM, our group reported iron over load and delayed physical growth [6]. In addition, in the same group of patients altered bone and mineral homeostasis (as indicated by hypocalcemia, hyperphosphatemia, vitamin D deficiency and altered parathyroid hormone activity) was identified with high frequency of pain and fractures in 12.5% of patients (submitted for publication). Both high and low levels of intact Parathyroid hormone levels (iPTH) were noted. However, the interpretation of the iPTH levels to assess both primary and secondary hyper and hypo functioning of the gland can only be made in association with calcium (Ca) and phosphorus (P).

With recently identified newer phenotypes of hyperparathyroidism including normocalcemic hyperparathyroidism (NCHPT), hypercalcemia with inappropriately normal iPTH and functional hypoparathyroidism (subjects who do not demonstrate secondary hyperparathyroidism (SHPT) despite vitamin D deficiency); interpretation has become more challenging. Isolated levels of biochemical parameter do not provide an indication of the existing functional abnormality in the parathyroid gland in patients [7]. Identification of parathyroid gland dysfunction poses a major challenge for physicians engage in managing patients with β-TM.

Harvey et al. used mathematical modeling to develop a multidimensional nomogram to provide a biologically sensitive estimation of PTH taking serum Ca and 25 hydroxy vitamin D (25OHD) that physiologically regulate PTH release. Comparison of estimated PTH (maxPTH) from the nomogram with actually measured plasma iPTH level can assist a clinician in differentiating different parathyroid diseases. This is especially helpful in differentiating newer phenotypes, especially when they do not conform to an easy diagnostic category [8].

The aim of this study was to determine the prevalence of different types of parathyroid dysfunction in transfusion dependent β-TM using biochemical markers and assess the difference between measured and maxPTH using nomogram by Harvey et al. for its application in clinical practice. The anthropometric measurements and bone and mineral abnormalities have been described previously in these patients [6].

## 2 Methods

### 2.1 Study setting, patient recruitment and data collection

Study setting and patient recruitment has been described previously [6]. Briefly, 367 diagnosed cases of transfusion dependent β-TM between 5 and 17 years of age were recruited from the outpatient clinics of a non-profit organization providing free of cost blood components to the patients with various chronic blood disorder. Study was approved by ethical review committee of Aga Khan University Hospital. Blood samples were collected in fasting to analyze the following parameters: vitamin D (25OHD), calcium (Ca), intact parathyroid hormone (iPTH), phosphorus (P), magnesium (Mg), zinc (Zn), creatinine (Cr), albumin (Alb), SGPT, urinary Ca and Cr.

### 2.2 Classification of subjects into clinical groups

Subjects were categorized into clinical groups based on corrected calcium, iPTH and 25OHD as follows:

1. Primary hypoparathyroidism: patients with clinical and biochemical evidence of hypoparathyroidism including ***low Ca and iPTH*** below the reference interval irrespective of 25OHD status (Ca<8.6 mg/dl, iPTH <15 pg/ml).
2. Sub-clinical hypoparathyroidism: includes patients with ***low iPTH and 25OHD, low and normal Ca levels*** (Ca ≤8.6–10.2, iPTH 15–65 pg/ml, 25OHD<20 ng/ml).
3. Patients with normal functioning parathyroid gland: ***Normal Ca and iPTH and optimal 25OHD*** (Ca 8.6–10.2, iPTH 15–65 pg/ml, 25OHD>20 ng/ml).
4. Secondary hyperparathyroidism: include patients with ***high iPTH with either Ca and/or 25OHD deficiency*** (Ca <8.6 mg/dl and/or 25OHD<20 mg/dl, iPTH >65 pg/ml

### 2.3 Application of PTH nomogram to calculate difference between maxPTH and measured PTH to identify hypoparathyroidism

We calculated patient specific upper limit of normal PTH (maxPTH) using equation developed by Harvey et al. [120 - (6 × calcium) - (0.5 × 25OHD) + (0.25 × age)], in our patients [8]. This equation calculates the expected PTH for a specific subject on the basis of total Ca, 25OHD, and age measured on the same day.

Subject specific maxPTH was used to calculate the difference from measured PTH (iPTH) to identify patients with hypoparathyroidism/parathyroid gland dysfunction in each group. Taking allowable error of plasma PTH as 33.43%, difference between maxPTH & iPTH greater than allowable error was calculated. This work has been reported in line with the STROCSS criteria [9].

### 2.4 Data analysis

The statistical analysis was performed using the Statistical Package of Social Sciences (SPSS) version 20. Data distribution was assessed by Shapiro-Wilk test for normality. Frequency was generated for categorical and medians with interquartile range (IQR) for continuous variables. Mann-Whitney *U* Test was performed to assess association of biochemical parameters amongst different clinical groups.

## 3 Results

### 3.1 Biochemical parameters of bone health in patients with transfusion dependent β-TM

Table 1 reflects, overall and hypo/hyper function and deficient/sufficient states as measured by low and high values for all analytes in 367 patients with transfusion dependent β-TM. Majority of the patients had vitamin D deficiency (78.2%), with only 21.9% having sufficient vitamin D levels.

**Table 1:** Biochemical parameters of bone health in children with transfusion dependent β-thalassemia major (n = 367).

Biochemical Parameters	Median (Q3-Q1)	Hyper/Hypo Functioning States	Frequency (%)	Median (IQR)
Intact-PTH (pg/ml)	52.7 (75.6–33.5)	Low < 15	16 (4.4)	11.4 (12.68–7.4)
		Normal 15–65	224 (61)	41.2 (52.9–30.5)
		High > 65	127 (34.6)	88.6 (116–74.6)
Vitamin D (ng/ml)	13.09 (18.7–8.56)	Deficient < 20	287 (78.2)	11.05 (14.5–7.67)
		Sufficient > 20	80 (21.8)	25.59 (29.6–21.97)
Corrected Calcium (mg/dl)	8.8 (9.4–7.96)	Low < 8.6	155 (42.2)	7.86 (8.21–7.09)
		Normal 8.6–10.2	212 (57.8)	9.3 (9.62–9)
Serum Phosphate (mg/dl)	5.1 (5.9–4.3)	Low	17 (4.6)	2.46 (2.68–2.37)
		Normal	216 (58.8)	4.69 (5–4.16)
		High	134 (36.5)	6.13 (7–5.7)
Serum Magnesium (mg/dl)	2.01 (2.18–1.84)	Low	52 (14.2)	1.51 (1.63–1.31)
		Normal	205 (55.9)	1.96 (2–1.87)
		High	110 (29.97)	2.3 (2.44–2.2)
Fractional Excretion of Calcium (FeCa) %	0.2 (0.5–0.1)	Normal < 1	334 (91)	0.2 (0.4–0.1)
		High > 1	31 (8.4)	1.3 (2.1–1.2)

Results are presented as median (Q3-Q1), and frequency (percentages).

### 3.2 Biochemical classification of patients

Table 2 shows the distribution of 367 patients, grouped into different groups based on their median Ca, 25OHD and iPTH blood levels with the median (IQR) levels of individual analytes in each clinical group.

**Table 2:** Classification into Groups of Parathyroid Dysfunction & Blood Biochemical Profile in Transfusion Dependent β-TM (n = 367).

Parathyroid Gland Status	n	Age years	iPTH (16–65 pg/ml)	25OHD (<20 ng/ml)	Serum Ca (8.6–10.2 mg/dl)	Serum PO4 (2.5–4.5 mg/dl)	Urinary FeCa <1%	Serum Mg (1.7–2.1 mg/dl)	Ferritin (ng/ml)
Primary Hypoparathyroidism	11	13 (15–12)	11.3 (12.6–7)	14.4 (24.5–10.1)	6 (7.4–4.7)	9.7 (10.6–7.9)	0.37 (0.75–0.09)	2 (2.1–1.9)	9105 (10126–4986)
Subclinical Hypoparathyroidism	192	11 (13–7)	40.4 (52.7–28.7)	10.7 (14.8–3.5)	8.38 (9.09–7.76)	5.1 (6.15–4.26)	0.003 (0.006–0.001)	1.96 (2.12–1.79)	4789 (6460–3255)
Normal Response for iPTH	39	9 (12–7)	44.2 (53.8–33.4)	25.8 (29.9–21.5)	9.6 (9.9–9.2)	4.9 (5.4–4.5)	0.27 (0.75–0.11)	2.1 (2.2–1.9)	3271 (6528–2643)
Secondary Hyperparathyroidism	125	11 (14–8)	88.6 (116–74.7)	13.6 (18.3–10.4)	9.1 (9.5–8.4)	5 (5.5–4.3)	0.21 (0.44–0.09)	2 (2.2–1.9)	5540 (7227–3541)
P value	–	0.079	<0.001	<0.001	<0.001	<0.001	0.06	<0.001	<0.001

Results are presented as median (Q3-Q1), and frequency (percentages). Where FeCa stands for fractional excretion of calcium, calculated by [(urine Ca × serum Cr) ÷ (serum Ca × urine Cr) × 100], normal FeCa <1%.

Majority (51%) of the patients had sub-clinical hypoparathyroidism followed by secondary hyperparathyroidism (34%). Among the 16 patients with low iPTH levels (Table 1), 05 were normocalcemic with median 25OHD levels of 12.5 ng/ml, suggestive of hypoparathyroidism.

Median Mg levels were within range, while high median P was present in all groups with highest level in patients grouped as primary hypoparathyroidism followed by subclinical hypoparathyroidism and secondary hyperparathyroidism group. Of note, is the high median P in the group despite normal response of parathyroid gland.

Median 25OHD levels were deficient in all other groups, but despite a similar vitamin D deficient state the response of the gland shows progressive worsening of parathyroid glandular function with advancing age and ferritin levels as is reflected by iPTH, in Table 2 in different groups.

Limited number (10.6%, n = 39) with normal response of iPTH were identified to have normal levels of corrected Ca, Mg, iPTH, and 25OHD levels, but P levels were high in 7 of these patients.

### 3.3 Application of PTH nomogram by Harvey et al

Patient specific max PTH was calculated using age, Ca and 25OHD status using nomogram developed by Harvey et al. Table 3 compares the median iPTH measured in the laboratory with the median expected PTH (maxPTH) calculated by the nomogram. The table also shows the number of individuals with difference in calculated ‘maxPTH’ and measured ‘iPTH’ was greater than the allowable error for PTH in different groups.

**Table 3:** Median Expected PTH (maxPTH) and Difference in maxPTH & iPTH in Different Groups of Parathyroid Dysfunction (n = 367).

Parathyroid Gland Status	n	iPTH pg/ml	maxPTH pg/ml	maxPTH & iPTH difference pg/ml	Difference ≥ Allowable Error
Primary Hypoparathyroidism	11	11.3 (12.6–7)	79.5 (87.3–67.1)	72.3 (76.5–54.5)	11 (100)
Subclinical Hypoparathyroidism	192	40.4 (52.7–28.7)	66.4 (73–60.9)	27.6 (42.1–12.3)	134 (69.8)
Normal Response for PTH	39	44.2 (53.8–33.4)	51.2 (53.5–49.2)	−5.2 (18 to −4.8)	13 (33.3)
Secondary Hyperparathyroidism	125	88.6 (116–74.7)	57.6 (65.2–57.6)	26.3 (−13.4 to −55.7)	53 (42.4)
P value	–	<0.001	<0.001	<0.001	–

Results are presented as median (Q3-Q1) and frequency (%). The maxPTH was calculated using Harvey et al. PTH nomogram [120 - (6 × calcium) - (0.5 × 25OHD) + (0.25 × age)]. Allowable error of plasma parathyroid hormone was 33.43% [reference: Westgard database of Desirable Specifications for Total Error (derived from Imprecision, Bias, intra- and inter-individual biologic variation) https://www.westgard.com/biodatabase1.htm. Accessed on 12/4/18].

Difference between maxPTH & iPTH was highest in primary hypoparathyroidism, followed by subclinical and secondary hyperparathyroidism. High values are predicted by nomogram for all three groups.

The median expected PTH in clinical group of secondary hyperparathyroidism at a given age, calcium and 25OHD is lower than the measured iPTH. Non-significant differences were observed in iPTH and maxPTH in the normally functioning gland.

## 4 Discussion

In this study, we identified 11 undiagnosed patients of hypoparathyroidism at median age of 13 years indicating need for earlier screening. The reason for this is inadequate chelation as is indicated by high ferritin. Most of thalassemia guidelines are from developed countries, where strict measures are taken to ensure adequate chelation, so they recommend screening for hypoparathyroidism from 16 years. In our data we observed that primary hypoparathyroidism developed from the age of 12 years (median 13 years), while average age of patients with subclinical hypoparathyroidism was 11 years. Most guidelines on thalassemia managment recommend screening for hypoparathyroidism at 16 years by assessing Ca & P; followed by PTH if low Ca or P is found. We propose that in developing countries screening for parathyroid dysfunction should start from 9 years onwards.

This is the first study, where we have use a unique classification for categorization of parathyroid disorders in a high risk population where the main cause of parathyroid gland deregulation is iron deposition. The varying biochemical manifestations require interpretation specific for each case. Simultaneous laboratory testing with relevant markers including Ca, P, iPTH and 25OHD is important for diagnosis, clinical correlation and interpretation especially in areas where vitamin D deficiency is endemic. Patients can easily be missed if the analytes are tested individually. We have previously reported usefulness of the simultaneous testing of markers using bone health screening panel in detecting newer phenotypes of primary hyperparathyroidism and validating by applying nomogram by Harvey et al. [7]. Further, in this study we have determined its utility in calculating expected parathyroid hormone levels to determine hypo functioning. We found obvious differences in the secretary capacity of parathyroid gland in the four groups for expected PTH.

Using upper and lower cut-offs of biomarkers we describe a phase that can be regarded as sub-clinical hypoparathyroidism or impending hypoparathyroidism where there is inappropriately normal PTH levels in response to either vitamin D deficiency or low Ca levels. This requires repeat testing to confirm for primary hypoparathyroidism as well as follow-up for monitoring. We also identified 5 cases in which Ca levels were normal with low PTH levels and vitamin D deficiency. A probable explanation could be intravenous infusion of calcium which is very frequently given to these patients when they present with tingling and/or twitching based on presumption that hypocalcemia is a cause without testing for calcium or PTH.

It is believed that parathyroid glands have large reserves. A biphasic chronological time course is described for hyperparathyroidism; in the first sub-clinical phase PTH levels are elevated but serum Ca is normal, and this phase remains sub-clinical because PTH levels are rarely measured with normal serum calcium concentration [10]. Third International Workshop on the Management of Asymptomatic Primary Hyperparathyroidism officially recognized and labeled this phase as Normocalcemic hyperparathyroidism in 2008 [11]. This phase is followed by the development of frank hypercalcemia and a symptomatic disorder with bone loss and kidney stones. Whether, the same time course exist for hypoparathyroidism, has not been established till to date. Cusano et al. reported prevalence of 1.1–1.9% normocalcemic hypoparathyroidism (NHYPO) in two unselected, non-referral community dwelling population from MrO and DHS [12]. NHYPO is not yet considered a real diagnostic category as data is not available. Sixty eight subjects were identified by Cusano with statistically significant differences in bone turnover markers (bone alkaline phosphatase and cTX) than in normal group. However, none developed overt hypoparathyroidism on follow-up and persistent disease was noted in only 2 out of 26 subjects.

Calcium phosphate product was >55 in 15%, increase in fractional excretion of calcium was also noted in 8.4% of patients, which is alarming and requires careful supplementation tailored to specific need of each patient, shown in Table 4. Unmonitored treatment with either Ca or vitamin D may end in altered calcium phosphate product and the risk of tumor calcinosis and nephrocalcinosis in patients [13]. Moreover, if hypoparathyroidism evolves than it is important to provide replacement with 1 alpha vitamin D in addition to cholecalciferol [14]. Abnormal levels for Ca, P and Mg (Table 1) are prevalent in this study. However, we did not find any association of Mg levels with PTH levels.

**Table 4:** Distribution of calcium phosphate product and fractional excretion of calcium in different groups.

Parathyroid Gland Status	n	Urinary FeCa		Serum Calcium × Serum Phosphate Product	
		Median (Q3-Q1)	frequency of >1%	Median (Q3-Q1)	>55
Primary Hypoparathyroidism	11	0.37 (0.75–0.09)	1	53.3 (62–44.4)	5
Subclinical Hypoparathyroidism	192	0.003 (0.006–0.001)	19	42.9 (51.2–35.1)	31
Normal Response for iPTH	39	0.27 (0.75–0.11)	5	45.5 (51.1–36.9)	8
Secondary Hyperparathyroidism	125	0.21 (0.44–0.09)	6	47.3 (53–41.2)	11
P value	–	0.06		<0.001	

Results are presented as Median (Q3-Q1) and Frequency. Where FeCa stands for fractional excretion of calcium calculated by [(urinary calcium/serum calcium) × (serum creatinine/urinary creatinine) × 100].

The primary end point of our study is to identify the prevalence of parathyroid hormone disorders in a high risk group of patients. The manifestations of abnormalities in patients with thalassemia patients is alarming and calls for the need for optimization of thalassemia management [15]. Our findings clearly highlights the need for using simultaneous measurement of markers for assessment of parathyroid activity. In addition, application of nomogram has shown beneficial results in our previous (7) and this study. This requires clinical validation for confirmation.

This is the first study in Pakistan that has addressed parathyroid gland dysfunction in thalassemia major and highlighted the importance of improper chelation leading to morbidity in this children. Findings are alarming and requires immediate attention for optimization of management. There is a strong need to determine our population specific guidelines for diagnosis and management of bone related problems. Over the past decades, management of patients with thalassemia has improved significantly. However, thalassemia major remains a common and serious hematological problem in Pakistan. There is dire need to address bone health issues in these patients in Pakistan.

## Provenance and peer review

Not commissioned, internally reviewed.

## Ethical Approval

Ethical Approval was provided by Ethical Review Committee of Aga Khan University Hospital (2305-Pat-ERC-12).

## Sources of funding

Study was funded by University Research Council, Aga Khan University (URC Project ID: 112012P&M.

## Author contribution

**Dr Hafsa Majid**, performed all analysis, prepared bibliography, literature search and participated in the writing of the manuscript.

**Dr Lena Jafri,** performed literature search, contributed to manuscript writing.

**Dr Sibtain Ahmed,** performed literature search, involved in patients examination, and in manuscript writing.

**Dr Jamsheer Talati,** contributed in critical analysis, write-up and review of the manuscript.

**Dr Bushra Moiz,** PI of the grant, involve in study design, data collection, analysis, write-up of the manuscript.

**Dr Aysha Habib Khan**, Co-PI & Bone Consultant on the grant, involve in study design, data analysis, manuscript write-up.

## Research registry number

https://clinicaltrials.gov/ct2/show/NCT03947632?term=NCT03947632&rank=1.

ClinicalTrials.gov Identifier: NCT03947632.

## Guarantor

Dr Bushra Moiz, Professor & Consultant Haematologist, Aga Khan University.

Dr Aysha Habib Khan, Associate Professor & Consultant Chemical Pathologist, Aga Khan University.
